# Immune‐dysregulation harnessing in myeloid neoplasms

**DOI:** 10.1002/cam4.70152

**Published:** 2024-09-10

**Authors:** Mohammad Jafar Sharifi, Ling Xu, Nahid Nasiri, Mehnoosh Ashja‐Arvan, Hadis Soleimanzadeh, Mazdak Ganjalikhani‐Hakemi

**Affiliations:** ^1^ Division of Laboratory Hematology and Blood Banking, Department of Medical Laboratory Sciences, School of Paramedical Sciences Shiraz University of Medical Sciences Shiraz Iran; ^2^ Institute of Hematology, School of Medicine, Key Laboratory for Regenerative Medicine of Ministry of Education, Jinan University Guangzhou China; ^3^ Regenerative and Restorative Medicine Research Center (REMER) Research Institute of Health sciences and Technology (SABITA), Istanbul Medipol University Istanbul Turkey; ^4^ Department of Immunology, Faculty of Medicine Isfahan University of Medical Sciences Isfahan Iran

**Keywords:** acute myeloid leukemia, myelodysplastic syndrome, myeloproliferative disorders, tumor‐infiltrating immune cells

## Abstract

Myeloid malignancies arise in bone marrow microenvironments and shape these microenvironments in favor of malignant development. Immune suppression is one of the most important stages in myeloid leukemia progression. Leukemic clone expansion and immune dysregulation occur simultaneously in bone marrow microenvironments. Complex interactions emerge between normal immune system elements and leukemic clones in the bone marrow. In recent years, researchers have identified several of these pathological interactions. For instance, recent works shows that the secretion of inflammatory cytokines such as tumor necrosis factor‐α (TNF‐α), from bone marrow stromal cells contributes to immune dysregulation and the selective proliferation of *JAK2V617F+* clones in myeloproliferative neoplasms. Moreover, inflammasome activation and sterile inflammation result in inflamed microenvironments and the development of myelodysplastic syndromes. Additional immune dysregulations, such as exhaustion of T and NK cells, an increase in regulatory T cells, and impairments in antigen presentation are common findings in myeloid malignancies. In this review, we discuss the role of altered bone marrow microenvironments in the induction of immune dysregulations that accompany myeloid malignancies. We also consider both current and novel therapeutic strategies to restore normal immune system function in the context of myeloid malignancies.

## 1 INTRODUCTION

**FIGURE 1:**
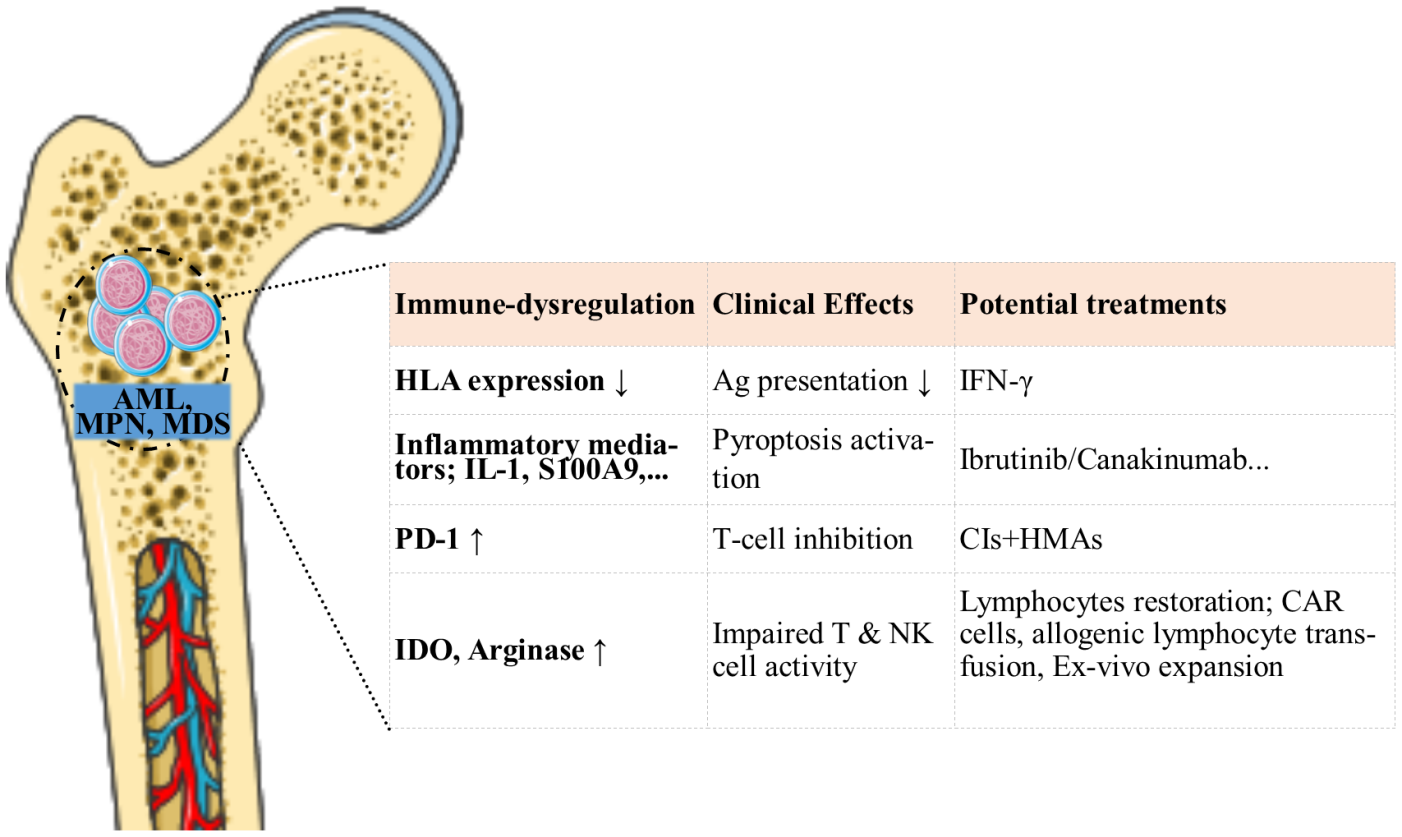
Myeloid malignancies are associated with key changes in the bone marrow immune microenvironment. By identifying more of these abnormalities, special treatments can be used to correct them and ultimately treat myeloid malignancies. The figure was made with the support of Dienst Medical Art (https://smart.servier.com). Ag, Antigen; HMAs, Hypomethylating agents; IDO; Indoleamine‐pyrrole 2,3‐dioxygenase.

The bone marrow microenvironment plays a crucial role in normal hematopoiesis and the development and progression of myeloid malignancies. Normal bone marrow niches are regulated by the interaction of various factors, such as stromal mesenchymal cells and their progeny, the vascular network, nerve endings, mature blood cells, extracellular matrix proteins, and bone cells. The main function of the bone marrow in a healthy person is to provide a nurturing environment and to help produce blood cells.^1^ In neoplastic myeloid diseases including acute myeloid leukemia (AML), myelodysplastic syndromes (MDS), and myeloproliferative neoplasms (MPNs), the functions of the immune system become dysregulated. Disruptions in the bone marrow microenvironment lead to uncontrolled clonal expansion of malignant cells. The molecular mechanisms underlying changes in the bone marrow environment and their effects on therapeutic approaches are not yet fully understood.^1^, ^2^ Interactions between stromal cells, immune cells, and neoplastic myeloid cells are dynamic and complex. Stromal cells such as fibroblasts and mesenchymal stromal cells secrete cytokines and other molecules to inhibit the anti‐tumor activity of the immune system, while antigen‐presenting cells, like dendritic cells (DCs) and macrophages promote malignant cells' growth and survival.^1^ Recently, several studies have focused on the link between chronic inflammation, aging, clonal hematopoiesis, and the development of hematological malignancies in the bone marrow microenvironment.^3^, ^4^, ^5^, ^6^ There is also significant mutational and phenotypic variability in myeloid malignancies.^4^ Therefore, it is difficult to make correct decisions regarding immunotherapy for myeloid malignancies. Despite these limitations, therapies targeting the immune‐dysregulated bone marrow microenvironment have been explored in recent years as promising methods for treating myeloid malignancies. Some immune‐targeting therapies, such as immune checkpoint inhibitors (CIs) and cytokines, may help restore immune system function to recognize and eliminate cancer cells. Targeting specific molecules involved in the development and progression of leukemic cells, such as FLT3 and epigenetic machinery, is effective in the treatment of myeloid malignancies due to their anti‐neoplastic and immunomodulatory effects.^7^ Potential passive immunotherapies for myeloid malignancies include tumor‐specific antibodies, cytokines, and adaptive cell transfer, while active immunotherapies include dendritic cell vaccines, allogeneic whole‐cell vaccines, checkpoint inhibitors, and oncolytic viruses, among others.^5^ This review aims to discuss and highlight the current information regarding the molecular mechanisms of alterations in the immunological bone marrow microenvironment in myeloid malignancies, as well as to provide insights into potential immunotherapies. A summary of main immune dysregulations and potential therapeutics is depicted in Figure 1.

## 2 IMMUNOLOGICAL TUMOR MICROENVIRONMENT IN MYELOID MALIGNANCIES, AN OVERVIEW

**FIGURE 2:**
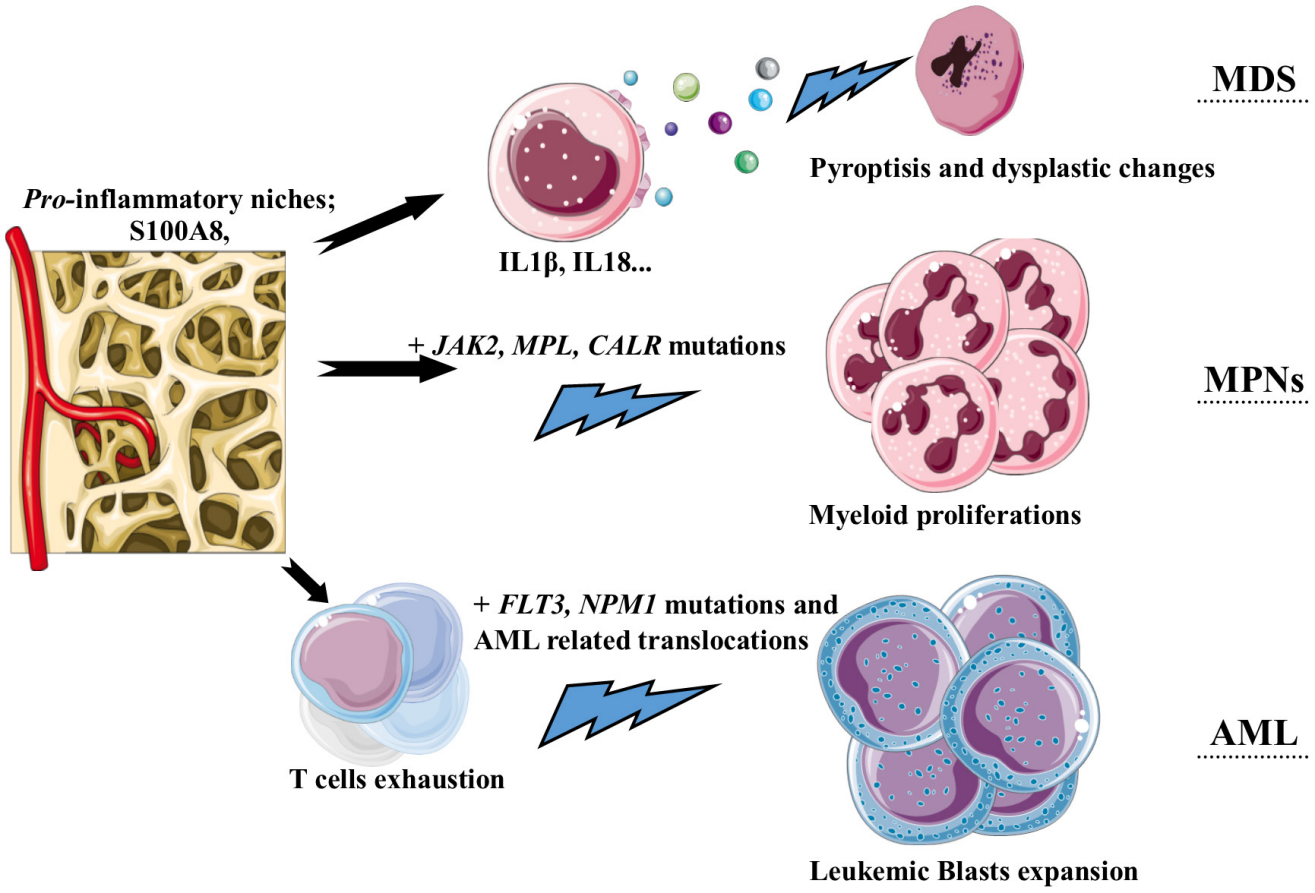
Current scheme of the role of the bone marrow inflammatory environment in the progression of myeloid malignancies. In MDS syndromes, inflammatory signals lead to a type of specific cell death called pyroptosis. Pyroptotic cells showed characteristic dysplastic changes associated with MDS neoplasia. In the presence of somatic mutations such as *JAK2V617F*, *MPL*, and *CALR*, an inflammatory microenvironment favors the development of myeloproliferative neoplasms. Inflammatory niches also lead to exhausted T and NK cells. In such situations, leukemic stem cells escaped immune surveillance and evolved into more aggressive AMLs. The figure was made with the support of Dienst Medical Art (https://smart.servier.com).

Although tumor immunology primarily focuses on the local immune responses within the tumor microenvironment (TME), it is important to acknowledge that these responses rely on continuous communication with peripheral organs. The bone marrow, blood, spleen, and draining lymph nodes together form an integrated immunological network that is in constant communication during the development of a myeloid malignancy.^5^ The progression of leukemia is influenced not only by the neoplastic cell compartment but also by the immunological milieu present within the TME.^8^ Interactions between bone marrow microenvironment and leukemia cells through a combination of adhesion molecules and soluble factors, play a crucial role in the development of myeloid malignancies. The immunological tumor microenvironment is well characterized for solid tumors but has recently gained attention for myeloid neoplasms. One of the major obstacles in conducting such studies in myeloid malignancies is that the immune cells themselves are part of the hematopoietic tissue and normal microenvironmental components of the bone marrow. However, due to the availability of powerful techniques such as multiplex immunohistochemistry, extensive studies have recently been conducted in this area.^9^, ^10^ The anticancer immune landscape consists of various elements. Innate immune cells, such as T cell subsets expressing γδ‐TCR, NK cells, myeloid suppressor cells, and phagocytes, are involved in the defense against leukemia. A consistent immune response to myeloid malignancies may be lacking due to factors like a low burden of malignant cell antigens, defects in antigen presentation, an increase in the number and activity of myeloid derived suppressor cells (MDSC), suppressive macrophages, an imbalance effector T cells, and regulatory T cell (Treg) (Treg cell dominance), T cell depletion caused by chronic inflammation, upregulation of immune checkpoint ligands and receptors, and production of immunosuppressive agents.^4^, ^5^, ^8^, ^11^, ^12^ Interestingly, in myeloid neoplasms there is a decrease in the cytolytic activity of lymphocytes of the innate immune system compared to lymphoproliferative disorders.^11^ Another important aspect of immune landscape in myeloid malignancies is the lowest tumor mutational burden among other neoplasms, which makes them unresponsive to PD‐L1 blockade immunotherapy.^13^ Advances in understanding the complex immune interactions associated with AML will support the development and application of personalized immunotherapeutic approaches.^14^ Recently, it has become clear that inflammation plays a major role in the development of myeloid malignancies, especially chronic types. Because of its vital role in the development of cancers, inflammation is now recognized as a hallmark of cancer.^15^ A clear example of the association between auto‐inflammation and myeloid neoplasia was recently described with the diagnosis of VEXAS syndrome.^16^ Each driver mutation in myeloid malignancies creates a specific inflammatory profile.^17^ Published studies have shown that certain inflammatory cytokines, such as TNFα and interferon‐α, play a selective role in the clonal expansion of myeloid neoplasms.^8^ These inflammatory cytokines activate positive feedback loops and transforming normal bone marrow niches into inflammatory microenvironments.^17^ The pathophysiological roles of the pro‐inflammatory bone marrow microenvironment in the development of myeloid malignancies are summarized in Figure 2. The role of a defective immune system and an abnormal immune microenvironment in the development and progression of myeloid malignancies is discussed in more detail in the following sections.

## 3 ACUTE MYELOID LEUKEMIA (AML)

Acute myeloid leukemia (AML) is a diverse group of acute hematologic malignancies derived from the myeloid lineage. The pathogenesis of AML involves various genetic alterations, including recurrent chromosomal structural variations and gene mutations, which typically determine the risk stratification of AML. Additionally, in recent years, epigenetic regulation and immune suppression have also been identified as playing roles in the pathogenesis of AML.^7^, ^18^, ^19^, ^20^, ^21^ Understanding the pathogenesis of AML is crucial for developing new treatment approaches and improving the accuracy of risk classification. Here, we focus on summarizing the immune suppression alterations that exist in the bone marrow microenvironment of AML, particularly focusing on alterations of T cells. We also review the immunotherapy approaches that have been utilized or show promise for future use.

### 3.1 Immune suppression and alterations in AMLs

Abundant studies have proven that the immune‐suppressive microenvironment has a significant impact on the development and prognosis of AML. CD8 T cells are at the center of anti‐tumor immunity, while γδ T cells, natural killer cells (NK), and other invariant NK T cells also play important roles in killing tumor cells. Numerical and functional abnormalities of these immune effector cells have been reported in the AML microenvironment. Furthermore, other abnormalities in regulatory immune cells such as regulatory Tregs, MDSCs, regulatory macrophage cells (Mφs), DCs, and MSCs also contribute to the construction of the immune suppressive microenvironment.^21^

The anti‐tumor function of NK cells is regulated by a balance of inhibitory and activating cell surface receptors. Multiple studies have found that AML patients often have low expression of activating receptors, like NK group 2D (NKG2D), natural cytotoxicity receptors (NCRs), and DNAX accessory molecule‐1 (DNAM‐1), while showing overexpression of inhibitory receptors, such as KIR2DL2/L3 and natural killer group 2A (NKG2A).^22^, ^23^, ^24^ In addition to the classic MHC‐I specific inhibitory receptors, other non‐MHC specific inhibitory receptors, like T cell immunoglobulin and ITIM domain (TIGIT), killer cell lectin‐like receptor (KLRG1), and programmed cell death protein 1 (PD‐1) are also over‐expressed on NK cells from AML patients.^25^, ^26^, ^27^ Additionally, the bone marrow microenvironment could hinder the cytotoxicity and proliferation of NK cells in AML through the release of immune‐suppressing cytokines, such as IL‐10, TGF‐β, IDO, and prostaglandin‐E2 (PGE2). These cytokines are secreted by leukemia blasts, MDSCs, and other cells.^22^, ^28^, ^29^, ^30^

MDSCs can suppress effector T cells through the release of arginase‐1 (Arg1), ROS, nitric oxide synthase 2 (NOS2), cyclooxygenase 2 (COX2), IL10, transforming growth factor beta (TGF‐β), and other cytokines. They can also mediate immunosuppression by upregulating Tregs. There is evidence showing that MDSCs accumulate in the peripheral blood (PB) and bone marrow of de novo AML patients.^29^, ^30^, ^31^, ^32^, ^33^ According to the literature, possible mechanisms related to MDSC‐mediated immune suppression in AML include high expression of Arg1, IDO, and V‐domain Ig suppressor of T cell activation (VISTA).^30^, ^34^

Macrophages (Mφs) are derived directly from monocytes under normal or inflammatory conditions.^35^ They can be broadly classified into two phenotypes: M1 (inflammatory) and M2 (regulatory). In blood cancers, leukemia associated macrophages (LAMs) are educated by the neoplastic bone marrow microenvironment to obtain leukemia‐supporting phenotype and mediate the progression of disease.^21^, ^36^, ^37^ The percentage of CD206^+^ M2‐like macrophages in the bone marrow was significantly elevated, and the level of infiltration of M2‐like macrophages positively correlated with poor outcomes for AML patients.^38^, ^39^ The molecular mechanisms related to the leukemia supporting phenotype polarization of Mφs in AML include low expression of the monocytic leukemia zinc finger (MOZ) and expression of Growth factor independence 1 (Gfi1).^39^, ^40^

### 3.2 T‐cell alterations in AML

Both αβ and γδ T cells can eliminate tumor cells, and their accumulation in the tumor microenvironment is usually correlated with better overall survival.^41^ One research that recruited 66 AML patients reported that high T cell percentages (>78.5% of total lymphocytes) in the bone marrow led to increased overall survival and leukemia‐free survival.^42^ Additionally, as reported by Ravandi et al.,^43^ a higher prevalence of CD3‐positive T cells in the bone marrow before the administration of therapy appeared to predict response to nivolumab in combination with induction treatment. In addition to changes in cell counts, various types of T cell dysregulation also occur in AML. These include impaired synapse formation, exhaustion, senescence, metabolic disturbances, and epigenetic changes. Immune synapse formation is the first step in T cell activation. However, in AML, the ability to form immune synapses and recruit phosphotyrosine signaling molecules is significantly impaired.^44^ In addition, the upregulation of immune check‐points (ICs), such as PD‐1, TIM‐3, CTLA‐4, LAG‐3, and TIGIT on CD8 T cells has been identified in de novo AML and relapsed and refractory AML (R/R AML).^45^, ^46^, ^47^, ^48^, ^49^ In relation to the cytotoxic cytokine secretion, Schnorfeil et al.^50^ found there were no functional defects in AML T cells. Meanwhile, Knaus et al.^48^ found that granzyme‐B expressing CD8 T cells were higher in de novo AML patients compared to the healthy control group. Additionally, Tang et al.^12^ have reported that the production of IFN‐γ by CD8 T cells from AML patients is impaired, but there was no reduction in IL‐2 and TNF‐α secretion. These different results may reflect the use of different stimulating agents for each experiment and the heterogeneous patient cohorts. Recently, several other T cell suppressive receptors expressed by AML blasts that inhibit T cell proliferation and function have been identified. These include leukocyte immunoglobulin‐like receptor B4 (LILRB4) and CD200. LILRB4 is an inhibitory immune checkpoint receptor that is selectively expressed on monocytic leukemic cells. It mediates the release of arginase‐1 (Arg1), which directly suppresses T cell proliferation and cytotoxicity.^51^, ^52^ CD200 is a type‐1a transmembrane cell‐surface glycoprotein. Coles et al.^53^, ^54^, ^55^ discovered that CD200 may inhibit T cell function by binding with CD200R expressed by T cells or by promoting Treg formation.

T cell senescence is a dysfunctional state characterized by the downregulation of costimulatory molecules CD27 and CD28, expression of senescence‐associated surface markers B3GAT1 (CD57) and KLRG1, an active metabolic state, and continuous secretion of pro‐inflammatory cytokines. Sergio Rutella et al.^56^ found that pre‐existing and chemotherapy‐induced senescent‐like T cells were correlated with a poor outcome in AML patients.

Tregs have been found to suppress the anti‐tumor function of T cells through direct cell‐to‐cell contact and secretion of inhibitory cytokines. Several studies have reported an increased number of Tregs in the peripheral blood and bone marrow of AML patients.^57^, ^58^, ^59^, ^60^, ^61^ Shenghui et al.^58^ also found that the accumulation of Tregs in the bone marrow is more significant than in the peripheral blood. These findings suggest that immune therapies targeting Tregs removal may enhance the remission rates of chemotherapy.

### 3.3 Immunotherapy Approaches in AML

Cytotoxic T cell plays a central role in the immune response against leukemia blast. Therefore, the targeted restoration of the T cell activity and adoptive transfer of gene‐manipulated T cells are generally considered for developing immunotherapeutic approaches.

Blocking CTLA‐4, TIM‐3, and PD‐1, or their ligands PD‐L1/PD‐L2, either alone or in combination with hypomethylating agents (HMAs) or chemotherapy agents, has been tested or is currently being tested in refractory/relapsed AML patients and those who have entered the remission phase but are at high risk of relapse. Some clinical trials have shown encouraging results, but more data from clinical trials are still needed to further explore which patient groups might benefit from ICIs.^62^, ^63^, ^64^, ^65^, ^66^

The only approved monoclonal antibody for treatment of AML is anti‐CD33 (gemtuzumab ozogamicin). Refractory/relapsed AML has a poor outcome. Gemtuzumab could induce a remission rate of 33% in these patients.^67^ New results demonstrate a significant decrease in the risk of relapse with gemtuzumab therapy in *NPM1*‐mutated AMLs compared to standard treatment (HR 0.65; 0.49–0.86; *p* = 0.0028).^68^ The primary results of anti‐CD70 monoclonal antibody therapy in elderly patients with AML are also encouraging. A single dose of cusatuzumab monotherapy followed by a combination therapy with HMA resulted in 10 out of 12 patients achieving complete remission, while 2 had partial remission.^69^

In vitro expansion of leukemia antigen‐specific T cells may have great potential for eliminating leukemia cells. However, difficulties in identifying and isolating of leukemia‐specific T cells have led to the development of antigen‐specific T cell receptor‐engineered T cells (TCR‐T) and chimeric antigen receptor T cells (CAR‐T). Unlike B‐cell lymphoma, AML has fewer surface markers on leukemia blasts that can be safely used. CD33 is expressed in up to 90% of AML blast cells but not in early pluripotent CD34^+^ hematopoietic stem cells, making it an attractive target. Several clinical trials using anti‐CD33 CAR‐T cells are currently ongoing. However, to date, only one study has reported on a patient with refractory AML who received autologous CAR‐T cell infusion. This patient experienced transient reduction in bone marrow blast cells along with cytokine release syndrome.^70^ CD123 is an ideal CAR‐T target for AML. One clinical trial involving six patients with refractory AML following allogeneic hematopoietic stem cell transplantation (HSCT) showed that after treatment with anti‐CD123 CAR‐T cells, one patient achieved a morphologic leukemic‐free state that lasted 2 months, two patients achieved complete remission (CR), and then proceeded to a second allo‐HSCT.^71^ In 2019, Yao et al.^72^ reported the achievement of CR with incomplete hematologic recovery in a patient who received a single infusion of donor‐derived CD123 CAR‐T cells as part of conditioning for haploidentical HSCT. While encouraging results have been shown, the myelotoxicity of anti‐CD33 and anti‐CD123 CAR‐T cells should be avoided due to their expression on healthy myeloid and myeloid progenitor cells. Other promising targets for AML CAR‐T cell development include Lewis Y (LeY), FLT3, CLL1, CD44v6, Folate Receptor β (FRβ), CD38, and CD7.^63^, ^73^, ^74^ TCR‐T is an approach to lyse tumor cells by genetically engineering T cells with a TCR that specifically recognize a leukemia antigen and MHC complex. Wilms “tumor 1” (WT1) is an overexpressed antigen in AML and MDS, with limited expression on normal CD34^+^ HSCs. Two clinical studies recruited patients with R/R AML to assess the efficacy of WT1‐TCR‐T. They found that the transferred T cells were well tolerated with minimal on‐target, off‐tumor toxicity. There were signs of anti‐leukemia activity, but no survival advantage was demonstrated.^75^, ^76^ Except for WT1, alternative intracellular antigens preferentially expressed by AML, such as PRAME,^77^ telomerase,^78^ HMMR/Rhamm,^79^ and the mutated form of NPM1^80^ have shown promise in clinical trials or preclinical research with engineered TCR‐T cells. Another approach is the use of bispecific antibodies to enhance the function of the patient's own T cells and overcome spatial barriers between effector T lymphocytes and leukemic blasts in the bone marrow. Bilantumomab is the first bispecific T cell engager (BiTE) immunotherapy that activates endogenous T cells by binding CD19 on acute B Lymphoblastic Leukemia (B‐ALL) cells and CD3 on T‐cells. This interaction leads to the formation of synapses between T cells and tumor cells, and ultimately resulting in the elimination of CD19^+^ cells. The success of bilantumomab spurred the development of various bispecific antibody constructs that link T cells to myeloid antigens in order to treat AML. CD33, which is widely expressed in AML leukemic stem and blast cells, has been particularly popular for constructing bispecific antibodies with CD3. There have been five different CD33‐CD3 bispecific antibodies that have been produced so far: AMG 330, AMG 673, AMV564, JNJ‐67571244, and GEM333. Each antibody has a unique construct aimed at extending its half‐life and increasing antigen affinity. In addition to CD33, other target antigens of interest in AML include CD123, CD47, CD70, FLT3, CLEC12A and CLL‐1.^81^, ^82^ Some of these targets are still under clinical trial investigation, while others have only been approved in preclinical studies. New results are expected in the future.

Several preclinical studies and clinical trials have provided insights into NK cell‐based immunotherapies, including adoptively transferred NK cells (CAR‐modified and cytokine‐induced), ICIs, and BiKE/tri‐specific killer cell engager (TriKE). GTB‐3550 TriKE is a cytokine immune engager that binds to CD16 on NK cells, CD33 on myeloid blasts and Interleukin 15 (IL‐15) between the two engager components. IL‐15 provides a self‐sustaining signal that activates NK cells and enhances their killing ability. In April 2021, Biopharma, Inc. launched the first‐in‐human GTB‐3550 TriKE Phase I/II clinical trial (NCT03214666) for the treatment of high‐risk myelodysplastic syndromes (MDS) and R/R AML. They reported a up to 63.7% reduction in bone marrow blast levels, resulting in clinical benefit, with no signs of cytokine release syndrome (CRS) or other observed toxicities.^83^ More detailed discussion of NK‐based immune therapy for AML has been reviewed by Xu et al.^84^

## 4 MYELODYSPLASTIC SYNDROMES (MDS)

### 4.1 MDS is an inflammatory disorder

Myelodysplastic syndromes are a diverse group of clonal stem cell malignancies characterized by increased apoptosis, ineffective hematopoiesis, resulting peripheral cytopenia, and an increased risk of transformation to AML.^85^ A thorough understanding of the pathogenesis of the hallmark characteristics of MDS has emerged from recognition of the role of reciprocal interactions of innate immune, cell‐intrinsic genetic alterations, and pro‐inflammatory signaling. The damage‐associated molecular pattern (DAMP) proteins S100A8 and S100A9 dimerize to create calprotectin, which directs a sterile inflammatory cell death called pyroptosis through autocrine and paracrine interactions.^86^ S100A9 leads to the expansion of mesenchymal niche cells and MDSCs. Additionally, cell‐intrinsic events directly stimulate S100A9 overexpression, providing a mechanism to induce pyroptotic cell death.^87^ Indeed, the extraordinary medullary expansion of MDSCs and pyroptosis are fundamental players in the disease that could be exploited therapeutically against MDS.

### 4.2 Pyroptotic cell death and innate immune system activation in MDS

Inflammasome‐directed pyroptosis is the basis for MDS phenotypes like macrocytosis, proliferation, and ineffective hematopoiesis.^88^ Multiple molecular mechanisms are involved in the precise regulation of inflammasome activation. Unlike apoptosis, noninflammatory caspase‐3 is responsible for mediating cell death. This process is initiated by multi‐portion inflammasome networks triggered by S100A8/A9 (alarmins or cytosolic DAMPS). Activation of TLR4 by key soluble mediators, leads to the increased expression of pro‐inflammatory cytokines like pro‐IL‐18, pro‐IL‐1β, and NLRP3.^88^ The interaction between NLRP3 and apoptosis‐associated speck‐like protein containing a caspase recruitment domain (ASC) following activation stimulates ASC polymerization (ASC specks). ASC specks act as a platform for the recruitment of pro‐caspase‐1 (through its CARD domain), which then undergoes autocatalytic cleavage. Active caspase‐1 produces IL‐1β and IL‐18 from their precursors, as well as activates the pore‐forming protein gasdermin D (GSDMD). GSDMD is a nonselective membrane pore‐forming protein involved in the release of ROS, IL‐18, IL‐1β, cations, and initiation of pyroptotic cell death.^89^, ^90^ The NLRP3 inflammasome activates pyroptosis‐mediated lytic cell death in the bone marrow, leading to ineffective hematopoiesis, death of healthy hematopoietic stem and progenitor cells (HSPCs), cytopenias, and β‐catenin‐induced proliferation of leukemic cells.^88^ This complex also contributes to hematopoiesis regulation.^91^, ^92^ The activation of pyroptosis by reciprocal interactions of cell‐extrinsic signals from alarmins and cell‐intrinsic genetic events, such as somatic gene mutations, is now recognized.

MDSCs are derived from bone marrow precursors usually as a result of perturbed myelopoiesis caused by various pathologies including a variety of cytokines and other molecules. In the BONE MARROW of patients with MDS, there is an accumulation of more MDSCs (Lin − HLA – DR – CD33+), which are the main effectors in the progression of cytopenia, accumulated.^93^ The binding of S100A8/A9 to the CD33 receptor promotes MDSCs, inducing further secretion of S100A8/S100A9, mobilization of granzyme‐containing granules, and production of immunosuppressive cytokines such as TGF‐β and IL‐10 to reduce effector T‐cell proliferation.^93^ MDSC‐derived S100A8/9 leads to autocrine and paracrine stimulation of pro‐inflammatory signals and further suppression of hematopoiesis and the innate immune system.^90^ Additionally, MDSCs have gained attention due to their association with poor prognosis resistance to chemotherapy and immunotherapy.^94^

High‐Mobility Group Box 1 (HMGB1), a ubiquitously expressed non‐histone DNA‐binding protein, shuttles between the nucleus and cytoplasm in nearly all eukaryotic cells. Upon cell activation or death, HMGB1 is released into the extracellular space, where it acts as an alarmin or damage‐associated molecular pattern (DAMP). Extracellular HMGB1 can activate various TLRs and is a confirmed activator of NLRP3, causing inflammation and pyroptosis.^95^ Recently, its role in limiting erythropoiesis in inflammatory‐mediated and sepsis‐mediated anemia of chronic disease has been elucidated.^96^, ^97^

### 4.3 Somatic mutations and inflammatory responses in MDS

Studies on the pathogenesis of 5q deletion (del(5q)) MDS have shown that genetic modifications can directly activate signals from the innate immune system signals. Haploinsufficiency of MiR‐145 and MiR‐146 in del(5q) leads to several consequences, such as overexpression of TRAF6 and IRAK1 and haploinsufficiency of TRAF‐interacting protein with forkhead‐associated domain B (TIFA).^98^

Somatic mutations affect both clonal selection and the immune microenvironment. Mutations in genes that modify the epigenetics (*DNMT3A*, *TET*‐*2*, *ASXL1*, and *EZH2*) or RNA splicing (*SF3B1*, *SRSF2*, and *U2AF1*) have been shown to increase pyroptotic fractions, pore formation, inflammation, and induction.^17^ These mutations also activate β‐catenin signaling and its target genes, which are downregulated by suppression of the NLRP3 inflammasome or NADPH‐derived ROS.^90^ As previously mentioned, somatic mutations in epigenetic modifiers create an inflammatory environment that enhances apoptosis. *TET2* mutations reduce the need for histone deacetylase 2 (HDAC2), which is associated with high levels of IL‐6.^99^, ^100^ *DNMT3A* mutation is more strongly correlated with increased IFN‐γ or TNF‐α through induction of HDAC9 expression.^101^ *ASXL1* mutations lead to increased NADPH oxidase, ROS, TLR4, and pyroptosis.^102^

Somatic mutations in the spliceosome compartments have been associated with innate immune dysregulation and inflammasome activation. Mutations in both *SF3B1* and *SRSF2* are linked to the hyperactivation of NF‐kB through downregulation of MAP3K7 and generation of a caspase‐8 isoform, respectively. Additionally, SRSF2 mutations result in elevated S100A8 and S100A9 alarmins.^103^, ^104^, ^105^, ^106^ Other somatic mutations, not involved in clonal hematopoiesis and driver mutations, lead to direct inflammatory manifestations and MDS.^90^ VEXAS syndrome (Vacuoles, E1 enzyme, X‐linked, Autoinflammatory, Somatic) is an adult‐onset systemic auto‐inflammatory disease associated with thrombosis that typically occurs in fifth to seventh decade of life.^16^ This syndrome results from somatic mutations in the gene *UBA1*, an X‐chromosome gene that provides instructions for making the ubiquitin‐like modifier‐activating enzyme 1. An increased risk of MDS has been reported in patients with VEXAS.^16^, ^107^ These data indicate that patients have an elevated risk for the developing myeloid and plasma cell neoplasms as well as symptoms of auto‐inflammatory diseases, and require monitoring for disease progression. The exact role of UBA1 somatic mutations in the induction of inflammatory flares should be clarified.

### 4.4 Therapeutic implications of targeting dysregulated immune pathways in MDS

To date, approved therapeutic interventions for MDS include lenalidomide for patients with transfusion‐dependent anemia due to lower‐risk MDS associated with del(5q) and hypomethylating agents (azacitidine and decitabine) for higher‐risk MDS.^90^, ^108^ The use of valid immune signatures for routine clinical research is expected to improve disease classification and patient outcomes.^109^ Given the importance of innate immune signaling and subsequent NLRP3 inflammasome activation in disease pathophysiology, the players involved in these pathways can be used as excellent pharmacological targets for future clinical practice.^108^ Novel therapeutic targeting of inflammatory/innate immune pathways in MDS including TLR signaling inhibitors, MDSC elimination, NLRP3 inflammasome inhibitors, IL‐1β inhibitors, Cas inhibitors, Wnt/β‐catenin antibodies, and PD‐1/PD‐L1 ICIs has been developed and has shown preliminary efficiency.^110^ Results of the phase Ib trial of the anti‐Tim3 (sabatolimab) combination with HMAs in 51 patients with high‐risk/very high‐risk MDS show an OS rate of 56.9%.^111^ This treatment protocol revealed a longer duration of clinical response (17.1 months) compared with HMAs alone treatments (10–15 months). Some clinical trials targeting inflammatory pathways are summarized in Table 1. Diagnosis of MDS could be challenging due to lack of diagnostic biomarkers in all cases. Pyroptosis and abnormal inflammatory responses can be harnessed as diagnostic biomarkers for MDS.^112^ These issues have been addressed by our published and ongoing works.^113^, ^114^

**TABLE 1:** Ongoing trials on innate inflammation pathway inhibitors in MDS.

Target biomolecules/ Therapeutic agent	Mechanism of action	Clinical trial phase/identifier	Disease state
TLR2/Tomaralimab	Anti‐TLR2 monoclonal antibody	Phase 2 completed/NCT02363491	Low‐risk MDS
TLR4/CX‐01	Disrupting the HMGB1 interaction with TLR4	Phase 1 completed/NCT02995655	High‐risk MDS
IRAK4/Emavusertib	Novel oral inhibitor of IRAK4 and FLT3	Phase 2 recruiting/NCT04278768	High‐risk MDS
NF‐kB/Bortezomib	Inhibition of NF‐κB activity by blocking proteasomal degradation of inhibitor of κBα (IκBα)	Phase 1 completed/NCT00580242	High‐risk MDS
NLRP3/Ibrutinib	Indirect inhibition of ASC	Phase 1 recruiting/NCT0255394, NCT03359460	High‐risk MDS
IL‐1/Canakinumab	Anti‐IL‐1 monoclonal antibody	Phase 2 recruiting/NCT0423915	Low‐risk MDS

Abbreviation: HMGB1, High‐mobility group box protein 1.

Immunosuppressive therapies, especially hourse anti‐thymocyte glubolin, have shown promising results in half of low‐risk MDS patients in a large international retrospective cohort study (involving 207 patients).^115^ The effectiveness of such treatments has already been proven in a small prospective study and a case report.^116^, ^117^

## 5 MYELOPROLIFERATIVE NEOPLASMS (MPNs)

### 5.1 Abnormal signaling leads to clonal myeloproliferation

According to the latest update from WHO classification, the main categories of MPNs are chronic myeloid leukemia (CML), polycythemia vera (PV), essential thrombocythemia (ET), and primary myelofibrosis (PMF).^118^ Common driver mutations that cause BCR‐ABL1‐negative MPNs are *JAK2V617F*, *CALR*, and *MPL*.^118^ The Janus kinase 2 gene (*JAK2*) is located on chromosome 9p24, and the *JAK2V617F* mutation occurs within its JH2 pseudokinase domain. Under normal circumstances, this domain has an inhibitory function that keeps the JAK2 protein in an inactive state. Ligand binding to cytokine receptors results in tyrosine phosphorylation of intracellular domains and auto‐phosphorylation of the JAK2 protein. This pathway leads to the activation of signaling mediators such as STATs (STAT5, STAT3, STAT1), ERK/MAP kinase and the PI3K/AKT/mTOR axis.^119^ The aberrant activity of signaling pathways following the *JAK2V617F* mutation disrupts the regulation of multiple transcriptional targets and normal cellular functions. These include the stimulation of proliferation and disruption of apoptosis in malignant clones by PIM kinases, impaired apoptosis, cytokine‐independent maturation, erythropoietin‐independent colony formation by BCL‐XL, and increased intracellular reactive oxygen species (ROS) following the PI3K‐AKT‐FOXO3a pathway signaling.^119^ ROS initiate inflammatory pathways that are crucial for the onset and progression of MPNs.^6^ Gain‐of‐function mutations in *MPL* and INDELs in *CALR* genes can promote JAK/STAT signaling, proliferation, and selective differentiation into the megakaryocyte lineage in a manner similar to the *JAK2V617F* mutation.^119^

### 5.2 Immune dysregulations in MPNs

Recent research has shown that MPN driver mutations are acquired many years before symptoms of disease appear.^120^ These mutations alone cannot cause malignant diseases and require additional factors, such as a weakened immune system. Since the bone marrow is the primary site for the development of myeloid malignancies, other related events also occur at this site. A variety of events, including pro‐inflammatory conditions, defects in antigen processing, and dysfunction of T lymphocyte are involved.^11^ In MPNs, due to the paracrine activity of malignant clones, healthy cells in the bone marrow environment exhibit abnormal functions that create optimal conditions for the growth of malignant cells. It has been shown that mutant CALR protein can trigger strong inflammatory responses and reduce the ability of normal immune cells to carry out phagocytosis and process antigen.^121^, ^122^ Recent studies indicate that, inflammatory cytokines play a crucial role in the pathogenesis of myeloproliferative malignancies.^17^, ^123^ They are produced by both neoplastic and stromal cells in the bone marrow. The inflammatory state in MPN bone marrow and overstimulation of mesenchymal stem cells (MSC) result in impaired communication between osteoclasts and osteoblasts in the niche.^124^ Defective activity of osteoclasts can lead to bone marrow fibrosis. Osteoclasts derived from mutant clone monocytes have both functional and morphological defects, showing abnormalities in size and resorption capacity.^125^ Neuropathy damage to sympathetic nerve fibers is another alteration that occurs in the MPN bone marrow microenvironment. This damage is due to the overproduction of IL‐1 beta from the *JAK2V617F* mutant clone. After such injuries, the Schwann cell and nestin + MSC content of the bone marrow is reduced.^35^ Hyper activated JAK–STAT signaling in neoplastic MPNs leads to the production of a variety of inflammatory cytokines.^126^ All three types of driver mutations in MPNs result in activation of the JAK–STAT pathway. Some inflammatory cytokines such as tumor necrosis factor‐α (TNF‐α) and interleukin‐1 beta (IL‐1β), have selective growth advantages for *JAK2V617F*+ clones.^127^, ^128^ TNF‐α is produced in myeloid cells following activation of the Toll‐like receptors (TLR). Negative feedback in TLR signaling is a critical step in regulating cytokine production. IL‐10 is a known inhibitor of TLR signaling. A study by Lai et al.^129^ showed that monocytes from MPN patients do not respond adequately to the anti‐inflammatory cytokine IL‐10. Therefore, aberrant TLR signaling and TNF‐α production result in a steady state of inflammation in patients with *JAK2V617F*+ MPNs. In addition to known factors that exacerbate inflammation in the bone marrow of patients with MPNs, it has recently been shown that the NLRP3 inflammasome may be part of this phenomenon.^130^ The NLRP3 inflammasome is a multiprotein part of the innate immune system that activates the generation of pathogen‐associated molecular patterns (PAMPs) and DAMPs in pathological conditions. Upon activation, pro‐caspase‐1 spontaneously cleaves to its functional form, converting pro‐IL‐1 and proIL‐18 to their mature forms.^131^ The NLRP inflammasome‐associated genes NLRP3, NF‐κB1, CARDS, IL‐1, and IL‐18 were expressed at elevated levels in MPN bone marrow samples, and their levels correlated with splenomegaly, *JAK2V617F* mutation, and leukocyte counts.^132^ Immune dysregulation and driver mutations have a two‐way correlation in MPNs. The *JAK2V617F* mutation can result in a reduction of T helper17 (Th17) cells, myeloid dendritic cells, and effector Tregs. While *CALR* mutations are associated with increases in innate lymphoid cell 3 (ILC3) and decreases in T helper 1 (Th1) cells.^121^ Neutrophil extracellular traps (NETs) are part of the innate immune system, resulting in an increase in cytoplasmic ROS levels of neutrophils and activation of myeloperoxidase, elastase, and protein arginine deiminase type 4 to promote chromatin decondensation.^133^ Wolach O et al. recently showed that neutrophil stimulation by ionomycin significantly increases NET formation in *JAK2V617F+* MPNs. They also found that advanced NET formation correlates with increased thrombosis in *JAK2V617F+* mice.^134^ It has previously been shown that MDSCs from MPN patients overexpress arginase‐1.^135^ Arginase‐1 converts l‐arginine to l‐ornithine and urea; therefore, an increase in arginase‐1 levels leads to l‐arginine depletion. l‐arginine deficiency can impair T‐cell differentiation and activity.^94^

### 5.3 Targeting immune system to improve defense against MPN malignancies

Ruxolitinib, an inhibitor of the tyrosine kinases JAK1 and JAK2, is the first drug approved for patients with moderate‐ and high‐risk PMF and PV who are unresponsive to hydroxyurea.^136^ Reducing spleen size and improving overall survival (OS) are the main effects of this inhibitor. These significant achievements have contributed to the anti‐proliferative and anti‐inflammatory effects of ruxolitinib.^137^ Treatment with ruxolitinib has a strong immunosuppressive effect and leads to a high risk of infection. More specific JAK2 inhibitors are expected to demonstrate a lower incidence of infection. Ruxoltinib only improves splenomegaly in 30–40% of PMF patients and does not have significantly impact on OS. Therefore, in recent years, efforts have been made to explore other compounds like BCL‐2 inhibitors, HMA agents, and other JAK inhibitors in conjunction with roxulitinib (NCT04562389, NCT03222609) and.^138^ In addition to being used as the first‐line treatment, roxulitinib has recently been used to control the progression of the blastic and accelerated phase of MPNs, and promising results have been obtained. One notable clinical features of these phases of MPNs is resistance to common treatments.^138^

Interferon‐alpha (IFN‐α) is a cytokine produced by various cells as part of the innate and cellular immune response and used as an immunomodulatory agent. Studies conducted over the years have shown that IFN‐α has diverse functions and could be effective in modulating disease symptoms. It has long been used to treat MPNs and other malignancies.^139^, ^140^ IFN‐α induces the expression of pro‐apoptotic genes such as caspase 4, caspase 8, tumor necrosis‐associated apoptosis‐inducing ligand (TRAIL), Fas/CD95 and the X‐linked inhibitor of apoptosis (XIAP).^139^ Other anti‐leukemia and immunomodulatory effects of IFN‐α include induction of cell differentiation, enhancement of macrophage, T‐lymphocyte and NK cell function, and enhancement of tumor antigen presentation.^141^, ^142^

## 6 DISCUSSION

Unlike other malignancies, normal innate immune cells are generated from the neoplastic microenvironment in myeloid neoplasms and exposed to dozens of known and unknown immune modulators. The story of the immune microenvironment is different for myeloid malignancies than for other neoplasms because the normal bone marrow microenvironment is the breeding ground for many cells of the immune system. In myeloid malignancies, the initial seeds of immunomodulatory events are sown by malignant cells. Such immunosuppressive effects occur through direct cell connections, production and secretion of cytokines and specific enzymes, and changes in the expression of antigen‐presenting molecules. Targeting each of these axes can form the basis of new treatments, but they have their own complexities.

Among myeloid malignancies, AML deserves special attention due to its poor prognosis. It is difficult to harness the abnormal immune environment in AML. Biologically, AML is a complex disease with intricate communications between blast cells and the bone marrow microenvironment. Impressive and innovative new methods are being tested to address these challenges. Conventional chemotherapy regimens or targeted therapies alone can have profound immunomodulatory effects.^48^, ^143^ Hypomethylating agents (HMAs) show promise in reactivating of immune anti‐leukemic responses.^144^ The therapeutic synergies of combining conventional chemotherapy (HMAs) with new immunotherapies in AML and high‐risk MDS are currently under investigation. Results are conflicting and further adjustments should be made considering biological complexities.^145^ One barrier to the interaction of CD3+ effector T lymphocytes with AML leukemic blasts is the spatial distance within the bone marrow microenvironment. An ingenious way to resolve this problem is to use BiTEs antibodies (NCT02152956, NCT02730312, NCT02520427). The application of CIs is a promising strategy to overcome the exhaustion of normal T cells in refractory/relapsed AMLs (NCT02996474, NCT02845297, NCT02775903, NCT03066648). Dysregulation of innate immune system cells can lead to disease progression, and future treatments should address these abnormalities. For instance, several studies have shown low expression of NKG2D, NCRs, and DNAM‐1as activating receptors and KIR2DL2/L3 and NKG2A overexpression as inhibitory receptors in AML patients.^146^ Adaptive NK cell transfer, in‐vitro expansion and monoclonal engineered antibodies that activate NK cell‐mediated antibody‐dependent cellular cytotoxicity (ADCC) are therapeutic strategies for harnessing NK cell immunity in AML.

**FIGURE 3:**
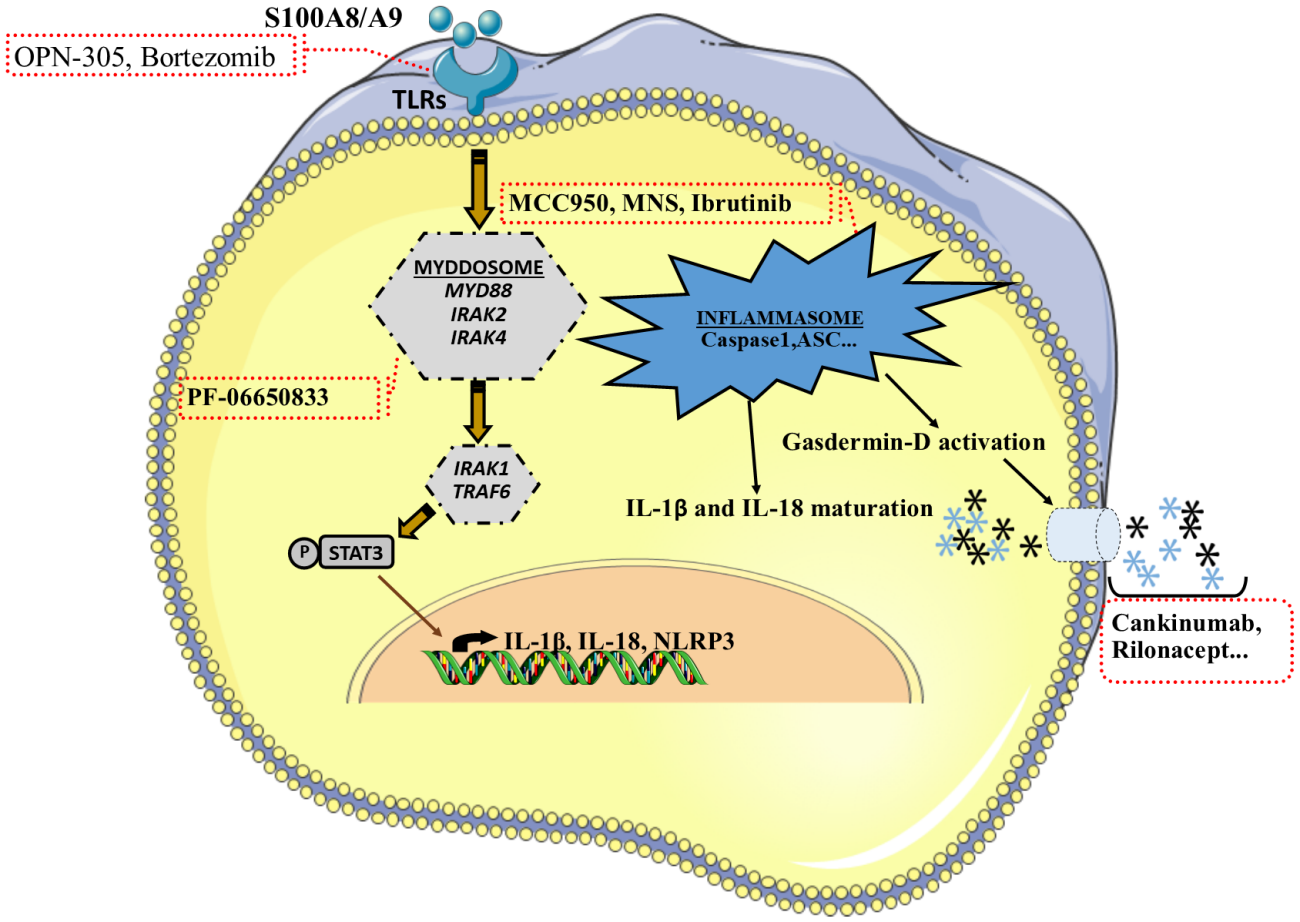
Potential clinical implications of inflammatory cascade inhibition in myelodysplastic syndromes. Pyroptosis is a cell death mechanism that is induced by inflammation and itself triggers an inflammatory milieu. At the onset of pyroptosis, surface TLR receptors are activated by S100A8/A9. The IRAK/MYD88 axis transmits TLR signaling to the nucleus through NF‐kB and phosphorylated STAT3. Eventually, this cascade led to gene expression of the inflammasome elements Gasdermin‐D, IL‐1 and IL‐18. By inflammasome formation and activation, pro‐gasdermin‐D, IL‐1β and IL‐18 will be cleaved and matured. Gasdermin‐D subunits formed a membrane pore. Mature IL1β and IL18 large cytokines could be released from the pyroptotic cells by these pores. As indicated by the red dashed boxes in the image, certain inhibitors of different stages of pyroptosis have potential clinical implications for MDS treatment. Some of these active ingredients are in clinical trials.^148^, ^149^, ^150^, ^151^, ^152^ The figure was produced with the assistance of Servier Medical Art (https://smart.servier.com).

Recently, it has been elucidated that inflammation plays a vital role in the development of MDS and MPNs. Therapeutic agents like ruxolitinib for *JAK2V617F+* MPNs, exert their effects mainly by suppressing the JAK–STAT pathway and subsequently alleviating of inflammatory niches. Upregulation of inflammatory cytokines through the NF‐κB pathway is involved in the pathogenesis of MDS. The demonstration of pyroptosis is a brilliant finding in the pathogenesis of MDS. Activation of the NLRP3 inflammasome in hematopoietic stem/progenitor cells leads to pro‐inflammatory pyroptotic cell death through caspase‐1. The result of inflammasome activation in the bone marrow is lytic cell death followed by ineffective hematopoiesis and sterile inflammation in BONE MARROW microenvironment.^88^ Modulation of the inflammasome activation pathway is considered a therapeutic targeting for MDS (see Figure 3). These inhibitors hold promise a better future for MDS, which currently have limited therapeutic options. The TLR‐IRAK‐NF‐κB axis is also involved in the pathogenesis of BCR‐ABL1 negative MPNs, and its therapeutic options may be useful for these entities.^123^ Immune cells such as MDSCs, NK cells, and dendritic cells, can also create an immune‐suppressive environment in MDS, and manipulating them therapeutically may be associated with improvements in clinical outcomes.^147^

## 7 CONCLUSION

Despite the central role of the bone marrow microenvironment in providing nourishment and immune modulation in myeloid malignancies, the specific players involved in the reciprocal interaction between leukemic cells and the immune system need to be identified. Recent advancements in understanding the pathologic mechanisms of immune system alterations in myeloid malignancies have enhanced our knowledge. A prime example of these advancements is the recognition of the inflammatory cytokines in immune modulation and the progression of MDS and MPNs. A better understanding of the key interactions within the bone marrow microenvironment requires the use of robust laboratory techniques. By elucidating the critical interactions that disturb immune system integrity in the bone marrow microenvironment, we can anticipate the development of more targeted treatments for myeloid malignancies. This review aims to summarize the current pathophysiologic findings and potential treatments for immune dysregulations in myeloid malignancies.

## AUTHOR CONTRIBUTIONS

**Mohammad Jafar Sharifi:** Conceptualization (equal); project administration (equal); visualization (lead); writing – original draft (equal); writing – review and editing (lead). **Ling Xu:** Writing – original draft (equal); writing – review and editing (equal). **Nahid Nasiri:** Writing – original draft (equal); writing – review and editing (equal). **Mehnoosh Ashja‐Arvan:** Writing – original draft (equal); writing – review and editing (equal). **Hadis Soleimanzadeh:** Writing – original draft (equal); writing – review and editing (equal). **Mazdak Ganjalikhani‐Hakemi:** Conceptualization (lead); funding acquisition (lead); project administration (equal); resources (lead); supervision (equal); writing – review and editing (equal).

## FUNDING INFORMATION

Thanks to TÜBİTAK for supporting the APC of the current work.

## CONFLICT OF INTEREST STATEMENT

The authors declare that the research was conducted in the absence of any commercial or financial relationships that could be construed as a potential conflict of interest.

## Data Availability

Not applicable.
